# Association of plasma sRAGE, but not esRAGE with lung function impairment in COPD

**DOI:** 10.1186/1465-9921-15-24

**Published:** 2014-02-25

**Authors:** Poornima Gopal, Niki L Reynaert, Jean L J M Scheijen, Casper G Schalkwijk, Frits M E Franssen, Emiel F M Wouters, Erica P A Rutten

**Affiliations:** 1 Department of Respiratory Medicine, Maastricht University Medical Center + (MUMC), Maastricht, the Netherlands; 2 Centre of expertise for chronic organ failure (Ciro+), PO Box 4080, Horn, the Netherlands; 3 Department of Internal Medicine, Maastricht University Medical Center + (MUMC), Maastricht, the Netherlands

**Keywords:** sRAGE, esRAGE, FEV_1_, COPD

## Abstract

**Rationale:**

Plasma soluble Receptor for Advanced Glycation End Product (sRAGE) is considered as a biomarker in COPD. The contribution of endogenous sRAGE (esRAGE) to the pool of plasma sRAGE and the implication of both markers in COPD pathogenesis is however not clear yet. The aim of the current study was therefore to measure plasma levels of esRAGE comparative to total sRAGE in patients with COPD and a control group. Further, we established the relations of esRAGE and total sRAGE with disease specific characteristics such as lung function and D_L_CO, and with different circulating AGEs.

**Methods:**

Plasma levels of esRAGE and sRAGE were measured in an 88 patients with COPD and in 55 healthy controls. FEV_1_ (%predicted) and FEV_1_/VC (%) were measured in both groups; D_L_CO (%predicted) was measured in patients only. In this study population we previously reported that the AGE N^ϵ^-(carboxymethyl) lysine (CML) was decreased, N^ϵ^-(carboxyethyl) lysine (CEL) increased and pentosidine was not different in plasma of COPD patients compared to controls.

**Results:**

Plasma esRAGE (COPD: 533.9 ± 412.4, Controls: 848.7 ± 690.3 pg/ml; *p* = 0.000) was decreased in COPD compared to controls. No significant correlations were observed between plasma esRAGE levels and lung function parameters or plasma AGEs. A positive correlation was present between esRAGE and total sRAGE levels in the circulation. Confirming previous findings, total sRAGE (COPD: 512.6 ± 403.8, Controls: 1834 ± 804.2 pg/ml; *p* < 0.001) was lower in patients compared to controls and was positively correlated FEV_1_ (r = 0.235, p = 0.032), FEV_1_/VC (r = 0.218, p = 0.047), and D_L_CO (r = 0.308, *p* = 0.006). sRAGE furthermore did show a significant positive association with CML (r = 0.321, *p* = 0.003).

**Conclusion:**

Although plasma esRAGE is decreased in COPD patients compared to controls, only total sRAGE showed a significant and independent association with FEV_1_, FEV_1_/VC and D_L_CO, indicating that total sRAGE but not esRAGE may serve as marker of COPD disease state and severity.

## Introduction

Chronic obstructive pulmonary disease (COPD) is a major cause of morbidity and mortality. Long term inhalation of noxious gases including cigarette smoke leads to airflow obstruction and emphysema in the lungs. On the other hand persistent systemic inflammation and oxidative stress are common features of this disease. Currently, soluble Receptor for Advanced Glycation End products (sRAGE) is considered as a potential biomarker for emphysema, based on data showing emphysema as independent predictor of decreased levels of sRAGE in the circulation of COPD patients [1,2].

RAGE is a transmembrane, multi ligand, pattern-recognition receptor that belongs to the immunoglobulin super family of cell surface receptors [3,4]. The human RAGE gene (*AGER*) consisting of 11 exons, produces membrane RAGE (mRAGE) which comprises an extracellular, a transmembrane, and cytosolic domain. Alternative splicing of the *AGER* gene leads to the formation of endogenous soluble RAGE (esRAGE) [5]. On the other hand, ectodomain shedding of mRAGE by metalloproteinase such as MMP9 and A disintegrin and metalloprotease (ADAM) 10 generates soluble RAGE (sRAGE) [6,7]. Soluble forms of RAGE found in the circulation can act as decoy receptors for Advanced Glycation Endproducts (AGEs), preventing binding of AGEs to cell bound full length RAGE and downstream activation of NF-kB [8].

Among many different extracellular ligands of RAGE, we previously examined levels of three different protein-bound AGEs in the plasma of COPD patients. In summary, N^ϵ^-(carboxymethyl) lysine (CML) was decreased, N^ϵ^-(carboxyethyl) lysine increased and pentosidine was not different in plasma of COPD patients compared to controls [9]. Literature survey shows that among several RAGE ligands investigated serum amyloid A (SAA), S100 protein A12 (S100A12) and High- mobility group protein B1 (HMGB1) were found to be increased in COPD patients compared to controls [10,11].

Although plasma sRAGE is considered as a biomarker for emphysema, no mechanisms have been proven to date that underlie this association. The most straightforward mechanism is that the lower plasma level of sRAGE is a reflection of the disappearance of pneumocytes which typically display very high RAGE expression [12]. A complicating factor is that it is assumed that the major soluble form of RAGE in the circulation is sRAGE. Recently however, a study identified an important contribution of esRAGE to total sRAGE as well as positive correlations of esRAGE to total sRAGE in BAL fluid and serum of asthmatics and COPD patients [13] Unfortunately, this manuscript did not report on differences in esRAGE levels between COPD patients and controls, or relations of esRAGE to disease markers.

In order to gain further evidence for plasma sRAGE as a potential biomarker in COPD, we measured plasma levels of esRAGE in patients with COPD and a control group. Furthermore, we established possible relations of esRAGE with different circulating AGEs, and disease specific characteristics such as lung function, D_L_CO and use of external oxygen. Circulating sRAGE levels were measured as well to identify the contribution of esRAGE to total sRAGE.

## Methods

### Study population

The study population included 88 moderate to severe COPD patients referred for pulmonary rehabilitation, and 55 healthy controls who were recruited in Ciro+, center of expertise for chronic organ failure, Horn, the Netherlands. Clinical history of COPD and the degree of disease severity were assessed according to the Global Initiative for Chronic Obstructive disease guidelines [14,15]. Exclusion criteria were history of tumor, diabetes, and an exacerbation of the disease for <4 weeks before blood draw. Control subjects were judged healthy by a standardized health questionnaire. This study was approved by the local medical ethical committee of Maastricht University Medical Center, the Netherlands and was supported by lung foundation Netherlands under AF2009 project no: 3.2.09.049.

### Patient consent

Patient consent: a written informed consent was taken from all study participants for the sake of publication.

The number of pack years (PY; the number of packs of cigarettes smoked per day divided by 20 multiplied by the number of years smoked) and the smoking status (never/ex-smoker) were recorded. People with 0 PY were considered never smokers, and who stopped smoking at least 1 year prior to recruitment was considered an ex-smoker. Lung function was determined by spirometry, and post-bronchodilator forced expiratory volume in 1 sec (FEV_1_) and forced vital capacity (FVC) were calculated from the flow-volume curve, and FEV_1_/VC was calculated and expressed as%. Diffusion capacity of carbon monoxide (D_L_CO) was assessed by using single-breath method (Masterlab®, Jaeger, Germany), expressed as %predicted and used as an indirect marker for emphysema [14,16,17]. Height and weight were measured in every participant and body mass index (BMI) was calculated (weight divided by height^2^ (kg/m^2^)). Use of long term oxygen treatment (LTOT) was recorded in the patients.

### Blood collection and determination of plasma markers

Blood was collected in an evacuated tube containing EDTA (Sherwood Medical, St Louis, Missouri, USA) and immediately centrifuged at 800 rpm for 10 min at 4°C. The plasma samples were subsequently stored at -80°C until analysis. Plasma esRAGE (B-Bridge international Inc., UK) and sRAGE (R&D systems, Minneapolis, USA) were measured by ELISA. Plasma protein-bound CML and CEL were measured by liquid chromatography tandem mass spectrometry [18] and pentosidine was measured by HPLC with fluorescence detection [19] and expressed per lysine concentrations. Plasma levels of high density lipoproteins (HDL), triglycerides, glucose, C–reactive protein (CRP) and creatinine were measured in an auto-analyzer (ABX Pentra 400, HORIBA ABX S.A.S, France). Glomerular filtration rate (GFR) was calculated using the Cockcroft-Gault formula [20].

### Statistical methods

SPSS (version 17, Chicago, IL) was used for data analysis. Variables with skewed distribution like esRAGE, sRAGE, CRP, FEV_1_, pentosidine and triglycerides were log transformed before further analysis. Comparison of characteristics between groups was performed by unpaired student’s *t* or Chi-square tests, for continuous or categorical data, respectively (Table 1). Spearman correlations were performed to establish the association of esRAGE and sRAGE with AGEs (Table 2) and other measured parameters (Tables 3 and 4). Uni- and multivariate linear regression analysis was used to investigate the association of plasma esRAGE and sRAGE with lung function, D_L_CO and LTOT. Primarily the parameters which showed a significant or a relevant (age and sex) association with esRAGE and sRAGE were included to build the regression models. Because of the co-linearity of the lung function parameters they were analyzed separately. All analysis were first adjusted for age, sex and GFR (models 1 and 2), next we evaluated the association of sRAGE and esRAGE with FEV_1_ or FEV_1_/VC (model 3), with D_L_CO (model 4), and with LTOT (model 5).

**Table 1:** General characteristics of COPD patients and controls

	**Never smoker controls, n = 11**	**Ex-smoker controls, n = 44**	**COPD, n = 88**
Age	58 ± 5.9	61 ± 5.4	63 ± 8^**E,N**^
Male, (n)%	(3) 27.3	(22) 50	(46) 52.3
Pack years^*^	0 ± 0	15.6 ± 13.3	35.0 ± 16.1^**E,N**^
Smoking status, (n)%^§^	-	-	Ex-smoker (64) 72.7 Current smoker (18) 20.5
BMI, kg/m^2^	26.9 ± 4.0	27.52 ± 4.0	26.1 ± 5.2
FEV_1_% predicted	114.5 (104.0-120.0	122.1 (112.0-133.0)	45.50 (32.3-61.0)^**E,N**^
FEV_1_/VC%	79.2 (74.6-82.3)	78.9 (75.2-82.5)	51.9 (38.3-63.3)^**E,N**^
D_L_CO% predicted	-	-	51.46 (42.4-73.19)
LTOT (n)	-	-	16
Triglycerides, mg/dl	92.2 (85.1-109.0)	93.0 (71.8-135.6)	100.1 (84.2-136.9)
HDL, mg/dl	69.1 ± 14.3	68.5 ± 20.5	75 ± 27
CRP, mg/L	0.6 (0.1-0.9)	0.7 (0.3-1.8)	3.4 (1.7-8.5)^**E,N**^
*Medication use*			
Long/short acting muscarinic receptor antagonist (n)%			63 (73.9)
LABA, (n)%			40 (45.5)
SABA, (n)%			23 (26.1)
LABA + inhaled corticosteroids, (n)%			54 (61.4)
Inhaled corticosteroid, (n)%			11 (12.5)
Oral corticosteroid, (n)%			17 (19.3)
N- acetylcysteine, (n)%			9 (10.2)
Statins, (n)%			22 (20.6)

Unless otherwise stated all data are expressed as mean ± SD or median (IQR).

*p-value* <0.05 between COPD patients and ex-smoking controls is noted as E, and between COPD and never smoking controls as N.

^*^indicates history of pack year smoked is missing for 34 patients; smoking stop date is available for this group.

^**§**^indicates smoking status of 6 patients were missing because of missing information on pack years smoked or smoking stop date.

Abbreviations used: BMI, body mass index; FEV_1_, forced expiratory volume in 1 second; HDL, high density lipoproteins; CRP, C- reactive protein; LABA, long-acting beta agonists; SABA, short-acting beta agonists.

**Table 2:** Correlations of sRAGE, esRAGE with AGEs in COPD

	**esRAGE**	**sRAGE**
CML	r = -0.034, *p* = 0.785	r = 0.321, *p* = 0.003
CEL	r = -0.220, *p* = 0.076	r = -0.11, *p* = 0.921
pentosidine	r = -0.032, *p* = 0.799	r = 0.106, *p* = 0.347

**Table 3:** Linear regression analysis of COPD patients with plasma esRAGE as dependent variable

**Univariate analysis**	**B**	**95% CI for B**	***p *** **value**
Age	0.029	0.006 to 0.052	0.016
Sex	-0.255	-0.637 to 0.127	0.187
Pack years	0.001	-0.012 to 0.014	0.925
BMI	0.022	-0.015 to 0.059	0.240
FEV_1_	0.007	-0.004 to 0.018	0.220
FEV_1_/VC	0.010	-0.002 to 0.022	0.108
LTOT	0.112	-0.418 to 0.642	0.674
CRP	0.015	-0.010 to 0.040	0.246
D_L_CO	0.012	-0.002 to 0.026	0.092
GFR	-0.010	-0.019 to 0.000	0.042
**Multivariate analysis**	**B**	**95% CI for B**	***p *** **value**
Model 1: Age + sex	-0.363	-0.754 to 0.028	0.068
Model 2: model 1 + GFR	-0.006	-0.015 to 0.004	0.271
Model 3: model 2 + FEV_1_	0.002	-0.011 to 0.015	0.784
Model 4: model 3 + D_L_CO	0.004	-0.004 to 0.026	0.157
Model 5: model 4 + LTOT	-0.011	-0.665 to 0.644	0.974
**Multivariate analysis**	**B**	**95% CI for B**	***p *** **value**
Model 1: Age + sex	-0.265	-0.638 to 0.107	0.068
Model 2: model 1 + GFR	-0.007	-0.019 to 0.006	0.287
Model 3: model 2 + FEV_1_/VC	0.007	-0.006 to 0.020	0.314
Model 4: model 3 + D_L_CO	0.009	-0.008 to 0.025	0.292
Model 5: model 4 + LTOT	0.009	-0.656 to 0.653	0.996

**B** is unstandardized coefficient.

## Results

### General description of COPD and controls

Table 1 describes the baseline characteristics of the study group. As expected, FEV_1_ was significantly lower in the COPD patients compared to the never and ex-smoking controls (p < 0.001). Based on the GOLD criteria 34 patients were categorized GOLD II, 39 as GOLD III and 15 as GOLD IV. COPD patients had smoked more PY compared to ex-smoking controls. Furthermore, no differences were observed in the plasma levels of HDL, triglycerides, creatinine and GFR between patients and controls. Lastly, CRP was found to be increased in COPD patients compared to both control groups (p < 0.001).

### Plasma esRAGE in COPD and controls

**Figure 1:**
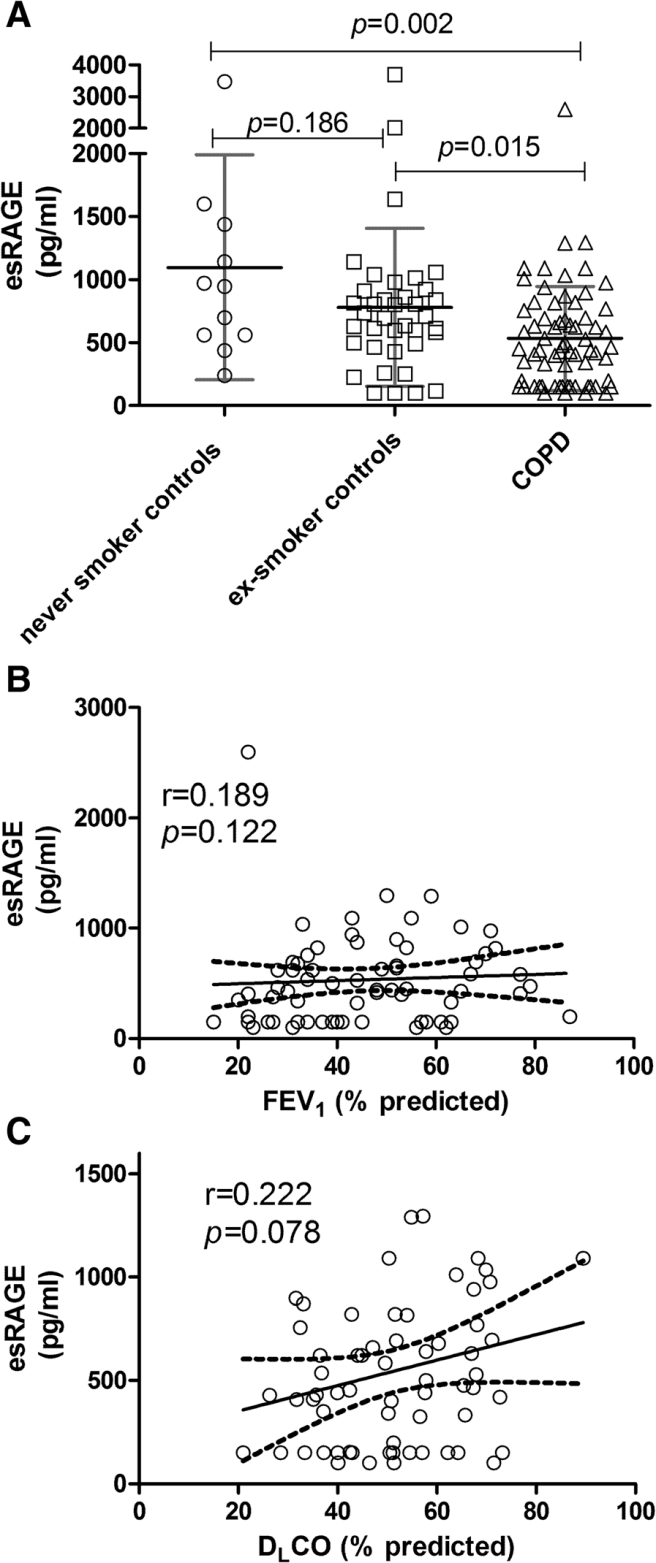
**Decreased plasma esRAGE levels in COPD patients compared to controls (A) are not correlated to FEV**_**1 **_**(B) or DLCO (C).**

Plasma esRAGE levels were significantly decreased in COPD patients compared to never and ex- smoking controls [never smoker: 1098 ± 893; ex-smoker: 781 ± 626; COPD: 534 ± 412, pg/ml, (Figure 1A)]. No effect of smoking was observed as the difference in plasma esRAGE levels between never smoking controls and ex-smoking controls, and between ex-smoking patients and current smoking patients was not significant (*p* = 0.186 and *p =* 0.340 respectively). esRAGE furthermore was not different between GOLD stages (GOLD II: 554.4 ± 340.8; GOLD III: 507.8 ± 468.6; GOLD IV:571.8 ± 393.2, *p =* 0.871) and plasma esRAGE levels did not show a significant correlation with FEV_1_ (Figure 1B), FEV_1_/VC (data not shown), and D_L_CO (Figure 1C) in COPD patients.

### Plasma total sRAGE in COPD and controls

**Figure 2:**
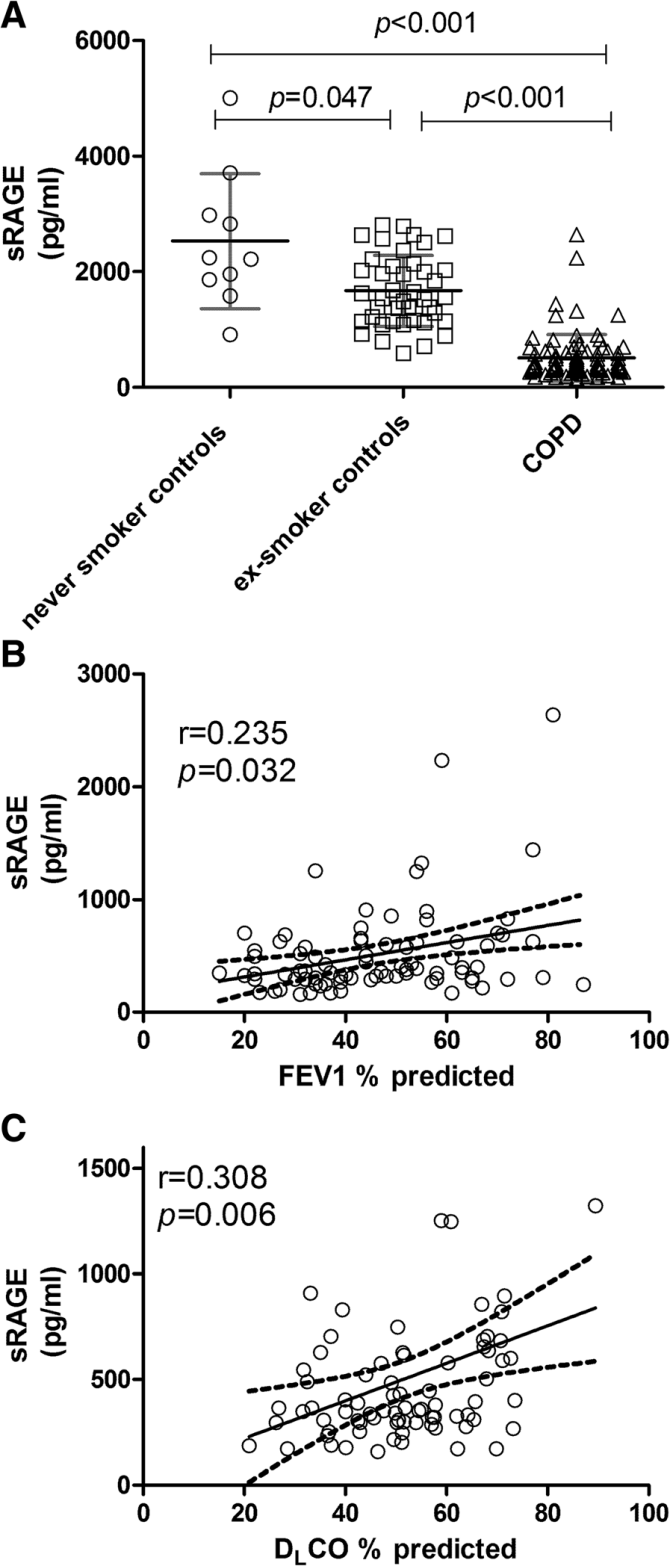
**Decreased plasma sRAGE levels in COPD patients compared to controls (A) are correlated with FEV**_**1 **_**(B) and DLCO (C).**

Plasma total sRAGE was also significantly decreased in COPD patients compared to the never and ex-smoking controls [never smoker: 2531 ± 1168; ex-smoker: 1671 ± 612; COPD: 512 ± 403 pg/ml, (Figure 2A)]. Plasma levels of total sRAGE were in addition found to be significantly lower in ex- compared to never-smoking controls (*p* = 0.047), indicating an effect of smoking per se. In COPD patients on the other hand, no significant difference in plasma sRAGE levels between ex-smokers and current smokers was observed (*p* = 0.503). As previously shown, sRAGE was significantly different between GOLD stages (GOLD II: 677.4 ± 576.8; GOLD III: 448.1 ± 241.7; GOLD IV:350.8 ± 144.9, *p* = 0.013) and plasma total sRAGE showed a positive correlation with FEV_1_ (r = 0.235, *p* = 0.032 (Figure 2B)) in COPD patients. A similar correlation was furthermore observed for FEV_1_/VC (r = 0.218, p = 0.047, Additional file 1: Figure S1), as well as for D_L_CO (r = 0.308, *p* = 0.006 (Figure 2C)).

### Correlations of esRAGE, sRAGE, AGEs and CRP

As expected, plasma esRAGE levels were positively correlated with total sRAGE levels. This correlation was found to be significant in the total study group (r = 0.436, p < 0.0001, Additional file 2: Figure S2), as well as in the COPD patients (r = 0.361, *p* < 0.001, Additional file 3: Figure S3b), and in healthy controls (r = 0.345, *p* = 0.016, Additional file 3: Figure S3a) individually.

No significant correlations were observed between plasma esRAGE levels and levels of AGEs in plasma. Plasma total sRAGE on the other hand was found to be positively correlated with plasma CML in COPD patients (r = 0.321, *p* = 0.003 (Table 2)), but not in control subjects. Plasma CEL and pentosidine were furthermore not significantly associated with total sRAGE levels. In addition, neither esRAGE nor total sRAGE showed a significant correlation with plasma CRP (data not shown).

### Association of plasma esRAGE and sRAGE with lung function, D_L_CO and LTOT

Table 3 represents the outcome of the univariate analysis taking plasma esRAGE as dependent variable. Only age demonstrated a significant positive and GFR a significant negative association with plasma esRAGE levels. Multivariate analysis performed as outlined in Table 3, introducing either FEV_1_ or FEV_1_/FVC after correcting for age, sex and GFR as common confounders, demonstrate that the decrease in plasma esRAGE levels in COPD was not determined by any of the disease severity markers examined.

Similar uni- and multivariate analyses were performed for plasma total sRAGE. Results of the univariate analyses in Table 4 indicate significant positive associations of total sRAGE with FEV_1_, FEV_1_/VC, and D_L_CO, and a negative association with LTOT. These variables were next used in multivariate analysis including age and sex as common confounders (model 1). Using this model, FEV_1_ showed a significant positive association with sRAGE [B: 0.010 (95% CI: 0.003 to 0.017); p = 0.006)]. When D_L_CO was introduced in the model the association remained with statistical significance (model 3, Table 4). However, when LTOT (as a dichotomous variable) was added to the model the direction of the association changed in conjunction with a stronger B value [model 2, B: -0.414 (95% CI: -0.781to -0.047); p = 0.028). A similar analysis was performed separately for FEV_1_/VC resulting in similar outcomes (Table 4).

**Table 4:** Linear regression analysis of COPD patients with plasma sRAGE as dependent variable

**Univariate analysis**	**B**	**95% CI for B**	***p *** **value**
Age	0.010	-0.005 to 0.025	0.190
Sex	-0.076	-0.325 to 0.173	0.547
Pack years	0.002	-0.006 to 0.010	0.630
BMI	-0.008	-0.032 to 0.015	0.480
FEV_1_	0.010	0.003 to 0.017	0.007
FEV_1_/VC	0.012	0.004 to 0.019	0.004
LTOT	-0.329	-0.640 to -0.017	0.039
GFR	-0.006	-0.011 to 0.000	0.069
CRP	-0.004	-0.019 to 0.011	0.584
D_L_CO	0.014	0.006 to 0.023	0.002
**Multivariate analysis**	**B**	**95% CI for B**	***p *** **value**
Model 1: Age + sex	-0.096	-0.345 to 0.153	0.444
Model 2: model 1 + FEV_1_	0.010	0.003 to 0.017	0.006
Model 3: model 2 + D_L_CO	0.012	0.002 to 0.022	0.017
Model 4: model 3 + LTOT	-0.414	-0.781 to -0.047	0.028
**Multivariate analysis**	**B**	**95% CI for B**	***p *** **value**
Model 1: Age + sex	-0.96	-0.346 to 0.153	0.444
Model 2: model 1 + FEV_1_/VC	0.012	0.004 to 0.019	0.004
Model 3: model 2 + D_L_CO	0.011	0.001 to 0.021	0.026
Model 4: model 3 + LTOT	-0.427	-0.787 to -0.066	0.021

**B** is unstandardized coefficient.

## Discussion

The present study demonstrates decreased levels of esRAGE and total sRAGE in plasma of COPD patients compared to controls. Importantly, in COPD, total sRAGE and not esRAGE showed positive associations with FEV_1_, FEV_1_/VC, and D_L_CO, and only sRAGE levels were negatively and independently associated with LTOT. Further, only total sRAGE levels were positively associated with CML, a major ligand of RAGE.

Most of the studies that report plasma sRAGE levels measured using a commercially available ELISA that detects total sRAGE, thus including esRAGE. Only one recent publication measured esRAGE separately in COPD. This study identified an important contribution of esRAGE to total sRAGE and positive correlations of esRAGE to sRAGE in BAL fluid and serum of asthmatics and COPD patients [13]. However, it did not report on differences in esRAGE levels between patients and controls, or relations to disease markers. Data from the present study confirm a moderate positive correlation of plasma esRAGE with total sRAGE levels and for the first time demonstrate decreased plasma esRAGE levels in COPD.

Two different mechanisms producing total sRAGE are by alternative splicing of *AGER* gene which results in secretion esRAGE, and by ectodomain shedding of membrane RAGE by MMP9/ADAM10 which leads to the secretion of sRAGE [21]. Both esRAGE and total sRAGE were observed to be lower in the circulation of COPD patients. Smoking per se seemed to affect total sRAGE levels as there was a significant difference between never and ex-smoking controls. This was however not the case for esRAGE (Figures 2A and 1A respectively), indicating that smoking affects shedding but not alternative splicing. Still it is important to further evaluate the effect of smoking as a cause for decreased sRAGE/esRAGE. Furthermore, the pattern of plasma sRAGE and esRAGE levels in the circulation of individual COPD patients and controls were found to be different. Based on these findings it is difficult to conclude whether alternative splicing or shedding or both are operational, since there is no literature on altered *AGER* gene expression and ADAM10 in the pulmonary/extra pulmonary compartment in COPD. However there is evidence in the literature of increased MMP9 protein levels in BAL of COPD patients [22,23], whereas an absence of difference in MMP9 levels and activity in the circulation of COPD patients was reported [24]. Further research is needed to determine whether altered gene expression, alternative splicing, and/or sheddases activity are responsible for decreased sRAGE levels in circulation.

Lowered levels of both esRAGE and sRAGE in the circulation of COPD patients could also be associated with the presence of functional single nucleotide polymorphisms (SNPs) in the *AGER* gene. Sequence variation studies have shown that there are 50 SNPs in 11 exons and 10 introns in the *AGER* gene. Among them rs2070600 [15] was associated with lower levels of plasma sRAGE in patients with diabetes and also in COPD [25,26]. Information regarding associations of circulating esRAGE levels with polymorphisms is limited. In type 2 diabetes it has been shown that rs2070600 and rs1800625 are associated with esRAGE levels in a Chinese cohort [27]. Given these genetic associations to circulating sRAGE levels in diseases affecting different primary organs and the contrasting increased pulmonary expression of RAGE in COPD patients, it remains questionable if lower systemic sRAGE levels indeed arise from altered pulmonary levels, and if so how [26,28]. There is one recent manuscript showing no decrease in plasma sRAGE level in patients with COPD with or without chronic heart failure [29], findings that are in contrast with many others, including our own showing decreased plasma total sRAGE levels in COPD [1,10,30].

Findings of the study presented here are in line with literature showing that plasma total sRAGE levels are associated with lung function in COPD [1,10,30,31]. In addition we evaluated whether comparable trends could be observed for esRAGE as both receptors have similar structures and functions, originating from the same gene through different mechanisms. In contrast to total sRAGE, we did however not find significant associations of plasma esRAGE with lung function parameters. Although the lack of CT data is a limitation of the current study, the finding that D_L_CO in most previous studies also correlated with plasma sRAGE levels, was replicated here as well. In addition we provide evidence that plasma total sRAGE levels are associated with the use of LTOT, even after adjustment for possible confounders and lung function. Again, such association with D_L_CO or oxygen use was not present for esRAGE. The mechanism of decreased systemic total sRAGE levels in COPD patients, and its association to alveolar damage is still not clear. From the results of the present study we can concluded that total sRAGE and not esRAGE resulting from splice variation of the *AGER* gene contributes to this association. Mechanisms involved in shedding of the receptor are likely involved, including via activation of sheddases like ADAM10 and MMP9, or via ligand engagement.

We investigated correlations of total sRAGE and esRAGE with measured plasma AGEs. The only significant correlation observed was between sRAGE and CML. RAGE ligands other than AGEs have been measured in the systemic circulation of patients with COPD. HMGB1, S100A12and SAA were found to be increased in COPD [10,11,32], Among these ligands, only SAA showed a negative association with sRAGE. We previously reported decreased plasma CML levels in COPD and we showed here that this is positively correlated with sRAGE. Accelerated formation and accumulation of AGEs is due to inflammation and oxidative stress [33]. However, we previously found that alterations in plasma levels of CML were independent of CRP, as a marker of inflammation [9]. With our previous [30] and present data we furthermore did not find significant correlations of either sRAGE or esRAGE with CRP. Only one study was able to demonstrate a negative association of sRAGE with CRP in COPD [10], but a clear relationship between sRAGE and inflammation and oxidative stress has not been found so far. Further investigations are clearly needed that assess the association of sRAGE/esRAGE with comprehensive panels of RAGE ligands as well as inflammatory markers in large cohorts of patients.

In contrast to the recent attention given to sRAGE in lung research, little is known about the role of esRAGE in pulmonary physiology and pathology. It has been shown that not only vascular endothelial cells express esRAGE, but also neuronal, thyroid follicular, hepatocytes and pancreatic beta cells could be a possible source of circulating esRAGE [34]. Plasma levels are in addition determined by excretion via the kidneys. Plasma esRAGE is known to be strongly affected by renal insufficiency [35]; in agreement we observe a significant negative correlation of esRAGE with GFR. The current study shows decreased esRAGE levels in COPD, but no associations with disease characteristics. The observed decrease in esRAGE levels in COPD is important for future investigations into molecular mechanism behind the decreased levels of total sRAGE demonstrated often already and proposed as a biomarker for the disease, but still warrants further confirmation. Secondly, to establish whether systemic sRAGE alterations are causatively related to the development of structural emphysema or a systemically reflective consequence further longitudinal research is warranted in non-obstructed cigarette smokers.

In summary, both esRAGE and sRAGE are decreased in the circulation of COPD patients compared to controls. Only sRAGE showed a positive association with lung function and D_L_CO, a negative association with CML and was affected by oxygen treatment in COPD. We conclude that, although these molecules are products of the same gene they may have different roles in the pathogenesis of COPD and that primarily total sRAGE is a candidate biomarker for COPD phenotypes.

## Competing interests

The authors declare that they have no competing interests.

## Authors’ contributions

ER and NR designed the study and ER provided the samples. PG and JS performed the analysis; CGS provided the equipment and reagents for AGE analysis. FF was a study physician. PG contributed for statistical analysis, PG, NR and ER drafted the manuscript, ER, NR, FF, EW, CGS and PG contributed to acquisition and interpretation of data. All authors read and approved the final version of manuscript.

## Supplementary Material

Additional file 1: Figure S1 Plasma sRAGE levels in COPD correlated with FEV_1_/VC. Click here for file

Additional file 2: Figure S2 Plasma levels of sRAGE and esRAGE in COPD correlated with each other. Click here for file

Additional file 3: Figure S3 Decreased plasma esRAGE compared to sRAGE in** (a) **controls** (b) **COPD patients. Click here for file
